# The SERRATE protein is involved in alternative splicing in *Arabidopsis thaliana*

**DOI:** 10.1093/nar/gkaa045

**Published:** 2020-01-20

**Authors:** Katarzyna Dorota Raczynska, Agata Stepien, Daniel Kierzkowski, Malgorzata Kalak, Mateusz Bajczyk, Jim McNicol, Craig G Simpson, Zofia Szweykowska-Kulinska, John W S Brown, Artur Jarmolowski

**Affiliations:** 1 Department of Gene Expression, Institute of Molecular Biology and Biotechnology, Adam Mickiewicz University, Poznan, Poland; 2 Department of Molecular and Cellular Biology, Institute of Molecular Biology and Biotechnology, Adam Mickiewicz University, Poznan, Poland; 3 Max Planck Institute for Plant Breading Research, 50829, Germany; 4 Biomathematics and Statistics Scotland (BioSS), James Hutton Institute, Dundee DD2 5DA, Scotland, UK; 5 Cell and Molecular Sciences, James Hutton Institute, Dundee DD2 5DA, Scotland, UK; 6 Division of Plant Sciences, University of Dundee at the James Hutton Institute, Dundee DD2 5DA, Scotland, UK

*Nucleic Acids Research*, 2014, 42(2): 1224–1244, https://doi.org/10.1093/nar/gkt894

**Figure 2.**
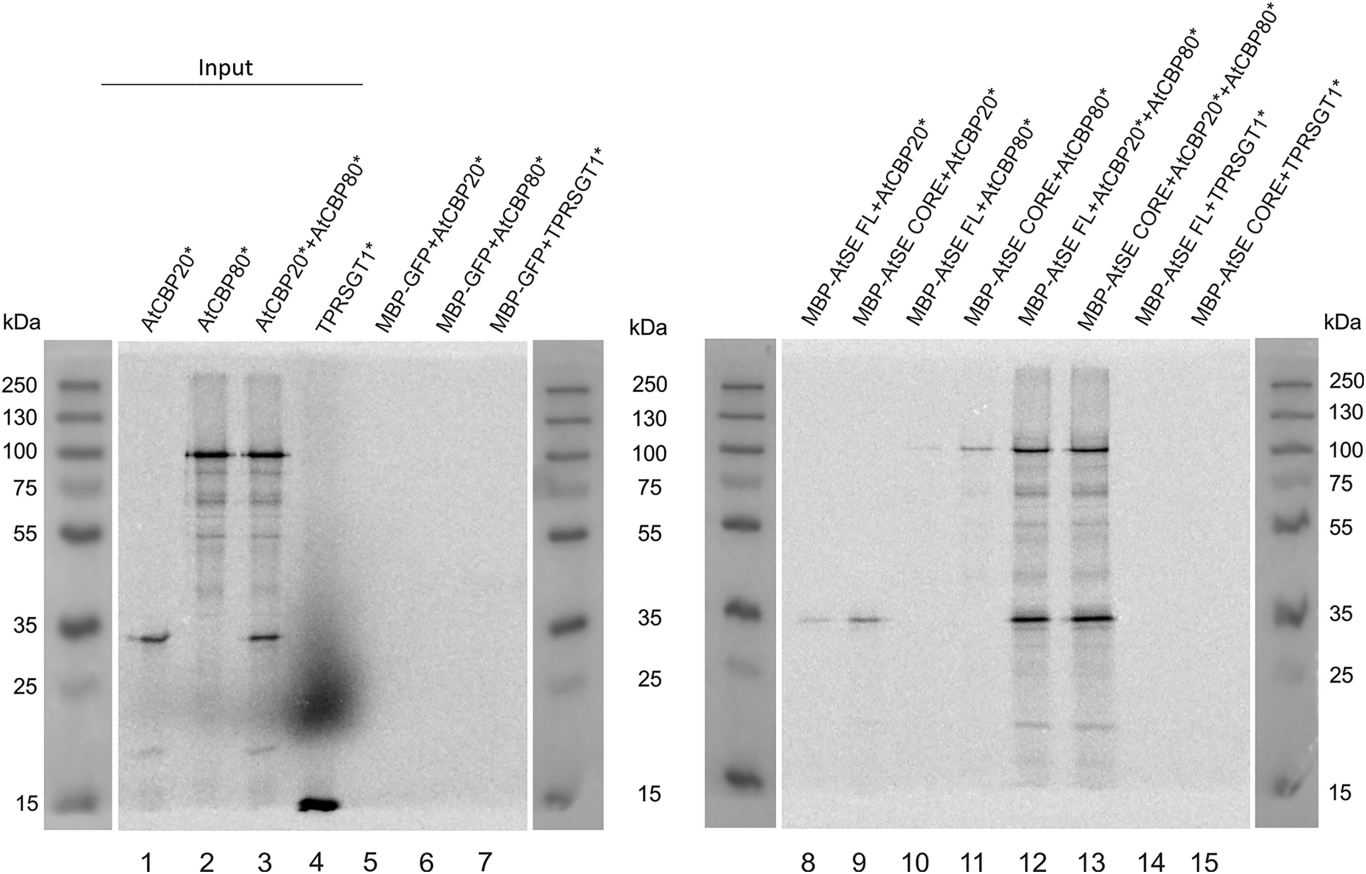
The interaction between full length (FL) AtSE or its core fragment (residues 194–543; AtSE core), and AtCBP20 and/or AtCBP80. AtSE FL, AtSE core and GFP proteins were overexpressed in bacteria as fusions to MBP. AtCBP20, AtCBP80 and TPRSGT1 (used as a negative control) were synthesized in the presence of [35S]-methionine (an asterisk in the protein name abbreviation means that the protein was labeled). MBP fusions and [35S]-methionine labelled samples were mixed in the combinations indicated. The resulting complexes formed were enriched on amylose beads, separated on a 10% SDS-PAGE gel and [35S]-methionine signal detected by exposure to an image analyzer. Inputs (lanes 1–4) represent one-tenth of the samples applied to amylose beads in the experimental lanes (5–15). PageRuler Plus Prestained Protein Ladder (Thermo Fisher) was used to monitor the size of the proteins analyzed.

The authors were recently made aware of anomalies in Figure 2. Before publication, the image was altered to cover unsightly cracks in the dried radioactive gel. In addition, the figure is a composite of 2 gels that were spliced together between lanes 5 and 6.

To confirm the results, the authors performed two fully independent repeats of this experiment with the same result, see new Figure 2 below. In the new figure, the results are presented on two separate gels. Additionally, the picture of PageRuler Plus Prestained Protein Ladder (Thermo Fisher) was overlaid to monitor the size of the proteins analyzed. The ladder was loaded on the same gels as the analyzed samples and the picture of markers was taken under day light. The samples were separated on 10% SDS-PAGE instead of 14% and inputs represent one-tenth of the samples instead of one-twentieth of the samples.

The results and conclusions of the article remain valid.

